# A Novel One-Dimensional Porphyrin-Based Covalent Organic Framework

**DOI:** 10.3390/molecules24183361

**Published:** 2019-09-16

**Authors:** Miao Zhang, Ruijin Zheng, Ying Ma, Ruiping Chen, Xun Sun, Xuan Sun

**Affiliations:** 1 State Key Laboratory of Crystal Materials, Shandong University, Jinan 250100, China; 201411920@mail.sdu.edu.cn (M.Z.);; 2 Key Laboratory of Functional Crystal Materials and Device, Shandong University, Ministry of Education, Jinan 250100, China; 3 Key Laboratory of Colloid and Interface Chemistry, Shandong University, Ministry of Education, Jinan 250100, China; 4 State Key Laboratory of Structural Chemistry Fujian Institute of Research on the Structure of Matter, Chinese Academy of Sciences, Fuzhou 350002, China

**Keywords:** covalent organic frameworks, one-dimensional structure, porous material

## Abstract

A novel one-dimensional covalent organic framework (COF-K) was firstly designed and synthesized through a Schiff-based reaction from a porphyrin building block and a nonlinear right-angle building block. The COF-K exhibited high BET surface area and narrow pore size of 1.25 nm and gave a CO_2_ adsorption capacity of 89 mg g^−1^ at 273K and 1bar.

## 1. Introduction

Covalent organic frameworks (COFs) are a class of porous crystalline materials constructed by rigid building blocks through covalent bonds [1,2,3]. It is fascinating that the COFs including the skeleton, pore size and pore shape can be predesigned and adjusted by modifying construction build units [4,5,6,7]. With this feature, COFs rapidly become a class of porous materials with wide applications, such as gas storage and separation [8,9,10,11,12], catalysis [12,13,14,15], optoelectronics [16,17,18,19,20,21], sensing [22,23,24,25], and drug delivery [22,26,27]. Since the first COF successfully synthesized by Yaghi and co-workers in 2005, a great deal of COFs has been fabricated over the past decade [28]. Generally, the topology design is achieved by planer or tetrahedral building blocks to form various crystalline, porous COFs structures. However, in spite of different structures, the topology of them only can be classified into two types: two dimensional and three dimensional. Due to the properties of COFs are determined by structure topology, for example, two-dimensional COFs with delocalization are favorable to electron conduction and three-dimensional COFs bearing highly specific surface area are beneficial to gas storage [29,30]. It is necessary to explore new topological structure COFs for its potential applications.

**Scheme 1:**
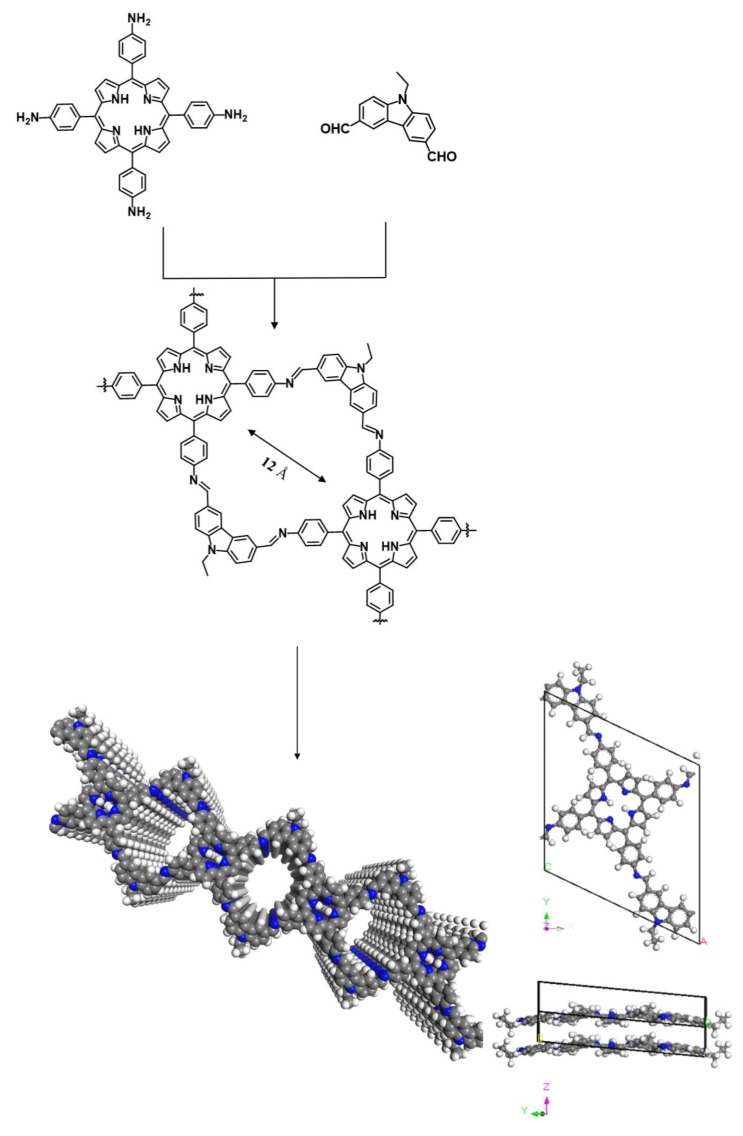
Synthesis route and structures of COF-K.

As for three-dimensional COFs, the structures extend in three directions to form three-dimensional networks and the structures extend in two directions to form two-dimensional plane for two-dimensional COFs. So, we define that the structures of one-dimensional COFs only extend in one direction to form one-dimensional line. COFs with one-dimensional topology have never been reported, and the performance of the one-dimensional COFs for CO_2_ adsorption remains to be disclosed. Porphyrin-based COFs have been extensively fabricated and studied due to the intriguing trait of their highly conjugated π-electron macrocycles and metal coordination sites. Specifically, for CO_2_ adsorption, the presence of basic pyrrole containing macrocyclic cavity is highly desired on account of the strong interaction with Lewis acid CO_2_ [31,32,33,34]. All the recorded porphyrin-based COFs have two- or three- dimensional topology and most of them use porphyrin as a C4 symmetrical building block to construct two-dimensional COFs with tetragonal pores. In this article, we report on a novel one-dimensional COFs based on a porphyrin building block named COF-K. To our knowledge, this is the first COF with a one-dimensional topology. Different from previously reported two-dimensional COFs is that each porphyrin connects four linear building blocks to form two-dimensional networks; each porphyrin links with four nonlinear right-angle carbazole building blocks to form one-dimensional linear topology in COF-K (Scheme 1).

## 2. Results and Discussion

COF-K was constructed from 5,10,15,20-tetrakis(4′-aminophenyl)porphyrin (POR) as the amino building block and 3,6-diformyl-9-ethylcarbazole (DFEC) as the aldehyde building block through a Schiff-based reaction. As a typical procedure, a mixture of POR and DFEC in o-dichlorobenzene/1-butanol/6 M AcOH (5:5:1 by volume) was heated in a sealed Pyrex tube at 120 °C for 72 h. Crystalline COF-K in the form of brown powder was obtained by Soxhlet extraction with tetrahydrofuran (THF).

**Figure 1:**
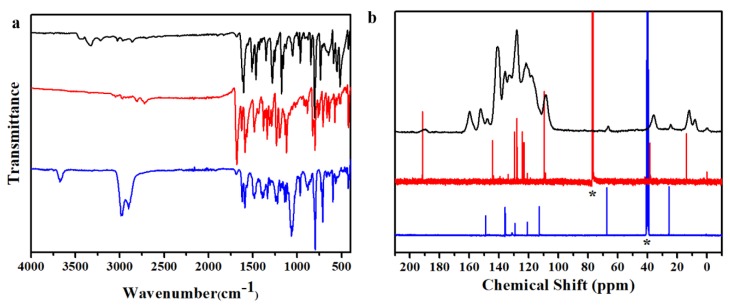
(**a**) FT-IR spectra of 5,10,15,20-tetrakis(4-aminophenyl)porphyrin (blue curve), 3,6-diformyl-9-ethylcarbazole (red curve) and COF-K (black curve); (**b**) ^13^C CP/MAS NMR spectra of COF-K (black curve), 3,6-diformyl-9-ethylcarbazole (red curve), 5,10,15,20-tetrakis(4-aminophenyl)porphyrin (blue curve). Note: * represents solvent peak.

The structure of the novel COF-K was confirmed by solid state ^13^C cross polarization/magic angle spinning nuclear magnetic resonance (^13^C CP/MAS NMR) and fourier transform infrared (FT-IR) spectra as shown in Figure 1 The FT-IR spectrum of COF-K appeared as a characteristic C=N stretching vibration at 1620 cm^−1^, which suggested the formation of COF-K. (Figure 1a, black curve) Furthermore, the disappearance of the N-H stretching vibration (3100–3400 cm^−1^) of POR and the C=O stretching vibration at 1680 cm^−1^ and Fermi double resonance at 2808 and 2722 cm^−1^ of DFEC in the FT-IR spectrum indicated that starting materials were completely consumed during the condensation reactions. The ^13^C CP-MAS solid-state NMR spectrum of COF-K and building blocks was shown in Figure 1b. The emerging characteristic signal of COF-K (Figure 1b black curve) at 160 ppm was the solid evidence of formation of the C=N based COF structure. All other peaks could correspond to building blocks correctly. Moreover, the peak at 190 ppm corresponding to carbon atom of carbonyl carbon in DFEC (Figure 1b, red curve) disappeared, which was an indication of the absence of starting materials. The morphology of COF-K was examined by scanning electron microscopy (SEM) and transmission electron microscopy (TEM), which showed a flake-like morphology in the SEM image and a strip-like layered-sheet arrangement morphology (Figures S1 and S2, ESI†). Thermogravimetric analysis (TGA) of the COFs showed high thermal stability up to 480 °C (Figure S3, ESI†).

**Figure 2:**
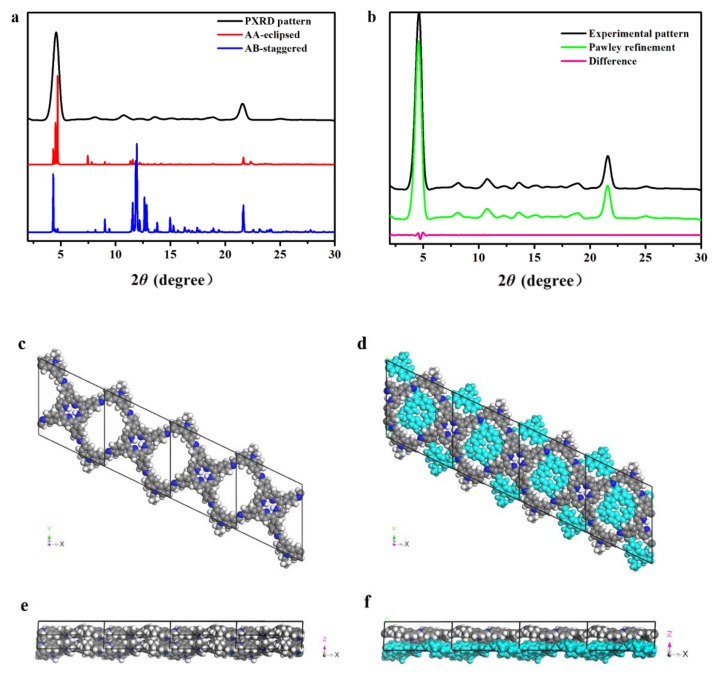
(**a**) Experimental PXRD pattern (black curve) and the simulated patterns according to AA-eclipsed (red curve) and AB-staggered (blue curve) stacking models; (**b**) Experimental PXRD pattern (black curve) and Pawley refinement (green curve) and difference between the experimental and refined patterns (pink curve) of COF-K; (**c**) Top view of the AA-eclipsed model structure of COF-K; (**d**) Top view of the AB-staggered model structure of COF-K; (**e**) Side view of the AA-eclipsed model structure of COF-K; (**f**) Side view of the AB-staggered model structure of COF-K.

To investigate the structure of COF-K, powder X-ray diffraction (PXRD) measurement and computer simulation were conducted. The PXRD patterns revealed that the COF-K powder was an ordered crystalline material though the PXRD pattern was relatively weak owing to lack of π–π interaction from layers. The intense peaks in the expected appeared at low-angle range. Furthermore, no peaks from the starting materials were detected, indicating the formation of pure crystalline COF-K materials (Figure S4). The spectrum of COF-K exhibited one strong peak at 4.62° and five weak peaks at 8.15°, 11.35°, 13.59°, 18.82° and 21.62°, which were assigned to (100), (210), (120), (300), (230) and (001) facets, respectively (Figure 2a, black curve). To illustrate the structure of the framework, Materials Studio 7 was used to build two possible one-dimensional models including eclipsed and staggered stacking (Figure 2c,d) and their simulated PXRD patterns (Figure 2a). The covalent bonds are bonding in xy plane to form one-dimensional line structure and non-bonding interaction π–π stacking is along the z axis as shown in Figure 2c or Figure 2e. The spectrum of the AA-eclipsed stacking model (Figure 2a, red curve) was in good agreement with the experimental PXRD pattern (Figure 2a, black curve) with slight differences that cannot be resolved by PXRD measurements, whereas the peaks of AB-staggered stacking model (Figure 2a, blue curve) showed large differences from the experimental PXRD pattern. We also built two possible two-dimensional eclipsed and staggered stacking models, analogous to that of common porphyrin-based COFs (Figure S5, ESI†). However, the simulated patterns of two structures were widely different from experimental pattern, indicating the different structure between two-dimensional models and the COF-K (Figure S6, ESI†). Pawley refinement was carried out for full profile fitting against the assumed models and figured out the unit cell parameters by using powder refinement module in Materials Studio 7, resulting in a suitable factor (R_wp_ = 1.97% and R_p_ = 1.02% after convergence) and reasonable profile differences and yielding the unit-cell parameters a = 21.705 Å, b = 22.765 Å, c = 4.128 Å, α = 85.43°, β = 89.86°, γ = 115.39° with a triclinic P1 space group. The Pawley refined patterns (Figure 2b green curve) matched well with the experimental pattern, as evidenced by their negligible difference (Figure 2b pink curve). Hence, we concluded that the COF-K was a one-dimensional eclipsed structure.

**Figure 3:**
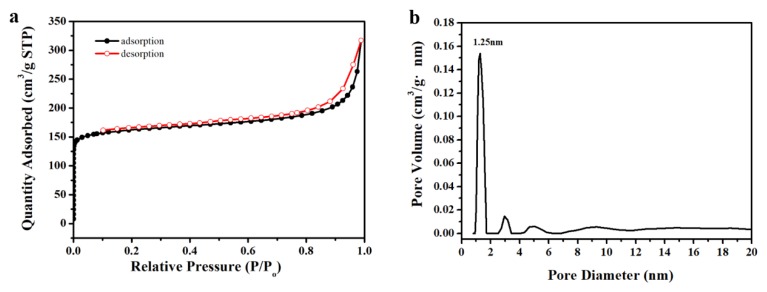
(**a**) Nitrogen adsorption-desorption isotherms of COF-K. (**b**) Pore-size distribution of COF-K.

To investigate the porosity and BET surface area of COF-K, the nitrogen adsorption-desorption isotherm was measured at 77 K after degassing at 120 °C for 12 h. As shown in Figure 3a, the isotherm of COF-K showed a Type-I isotherm feature according to the IUPAC classification and a steep increase under low pressure region (P/P_0_ = 0–0.01), indicating its microporous nature [35]. The Brunauer–Emmett–Teller (BET) surface area of COF-K was calculated to be 626 m^2^ g^−1^. The pore size distribution was also calculated by using the nonlocal density functional theory model (NLDFT), resulting in a pore diameter of 1.25 nm, which matched well with the theoretical value. Among two-dimensional porphyrin-based [4+2] COFs, for example, COF-366 (2 nm) [30], COF-367 (2.5 nm) [36], H_2_P-COF (2.5 nm) [29], TT-por COF (2.5 nm) [37], DmaTph (1.8 nm) [38], the minimum pore size was given a 1.8 nm. However, the pore size of COF-K was smaller than 1.8 nm, which was another evidence that the topology of COF-K could not be common two-dimensional structure.

Small pore size and pyrrole containing macrocyclic cavity were beneficial for CO_2_ adsorption. The CO_2_ adsorption capacity of COF-K reached 89 mg g^−1^ at 273K and 1bar (Figure S7, ESI†), which was higher than that of two-dimensional porphyrin based COFs, such as [HO]-H_2_P-COF(63 mg g^−1^,S_BET_ = 1284 m^2^ g^−1^) [39], [C=C]-TAPH-COF(51 mg g^−1^,S_BET_ = 680 m^2^ g^−1^) and [HO]-TAPH-COF(62 mg g^−1^,S_BET_ = 1056 m^2^ g^−1^) [40] and several non-functionalized COFs, for example, COF-5 (59 mg g^−1^, S_BET_ = 1670 m^2^ g^−1^) [10], ILCOF-1 (60 mg g^−1^, S_BET_ = 2723 m^2^ g^−1^) [41], COF-103 (76 mg g^−1^, S_BET_ = 3530 m^2^ g^−1^) [10]. Such a high capacity attributed to smaller pore size and pyrrole macrocyclic cavity instead of surface area [34,42].

## 3. Materials and Methods

### 3.1. Measurements

Fourier transform infrared (FT-IR) spectra were collected in KBr pellets with 2 cm^−1^ resolution using a Bruker ALPHA-T. Powder X-ray diffraction (PXRD) measurements were carried out on a Bruker D8 ADVANCE X-ray with a Cu-Kα sealed tube (λ = 1.5406 Å) from 2θ = 2° to 30° with 0.02° increment. Field emission scanning electron microscopy (FE-SEM) was performed on a *Hitachi* SU 8010 with accelerating voltage of 5.0 kV. The samples were sputtered with Pt (nano-sized film) prior to imaging by a SCD 040 Balzers Union. Transmission electron microscopy (TEM) images were obtained on a JEOL JEM-2100F electron microscope operated at 100 kV. The samples were prepared by dropcasting the sample from ethanol on copper grids. Thermogravimetric analysis (TGA) were taken on a Perkin Elmer Diamond by heating the samples at 10 °C min^−1^ to 1000 °C in an air atmosphere. The Brunauer–Emmett–Teller (BET) surface area and gas adsorption measurements were carried out at 77K with Micromeritics ASAP 2020 after being degassed at 100 °C for 12 h. ^1^H NMR spectra were recorded on Bruker AVANCE300, where chemical shifts were dependent on a residual proton of the solvent as standard. Solid-state ^13^C CP/MAS NMR measurements were measured on Bruker AVANCE III HD at a MAS rate of 5 kHz and a CP contact time of 2 ms.

### 3.2. General Materials and Methods

Pyrrol, p-nitrobenzaldehyde, phosphorus oxychloride, N-alkyl carbazole were obtained from Aladdin. Propionic acid, acetic anhydride, conc. HCl, SnCl_2_·2H_2_O, NH_3_·H_2_O were obtained from Sinopharm Chemical Reagent Limited Company. Pyridine, THF, n-hexane, dimethylformamide, 1,2-dichloroethane, chloroform, Tianjin Fuyu Fine Chemical Limited Company. All other reagents and solvents were commercially available and used as purchased without further purification. Soxhlet extraction was conducted through Soxhlet apparatus with THF.

Molecular modeling and Pawley refinement were implemented using Reflex, a module in *Materials Studio 7*. Unit cell parameters were first manually calculated from the observed PXRD peak positions using the Bragg equation. We utilized Pawley refinement to optimize the lattice parameters iteratively until the R_wp_ value converges. The pseudo-Voigt profile function was used for whole profile fitting and Berrar–Baldinozzi function was used for asymmetry correction during the refinement processes. The final R_wp_ and R_p_ values were R_wp_ = 1.97% and R_p_ = 1.02%.

### 3.3. Synthesis of 5,10,15,20-Tetrakis(4′-Nitrophenyl)Porphyrin

A 250-mL three-neck round bottom flask was contained the mixture of propionic acid (110 mL) and acetic anhydride (5 mL), p-nitrobenzaldehyde (1.4 g, 9.27 mmol) was heated to 173 °C, then freshly distilled pyrrole (0.64 mL, 9.27 mmol) was added dropwise. After dropping, the solution was stirred at 173 °C for 1 h. After the reaction completed, the dark violet precipitate was collected by filtration and washed with methanol. The crude product was dissolved in 100 mL pyridine and refluxed with stirring for 4 h, after cooling to room temperature, the product was collected by filtration to give 5,10,15,20-tetrakis(4′-nitrophenyl) porphyrin as violet powder (0.98 g) in 13% yield. The product was used directly for the next step without further purification. ^1^H NMR (CDCl_3_, 300 MHz): δ (ppm)–2.80(s, 2H, NH), 8.33 (d, 8H aromatics), 8.606 (d, 8H, aromatics), 8.755 (s, 8H, H-pyrrole).

### 3.4. Synthesis of 5,10,15,20-Tetrakis(4′-Aminophenyl)Porphyrin

The compound was prepared according to the literature. [43] A mixture of 5,10,15,20-tetrakis(4′-nitrophenyl)porphyrin (1.2 g, 1.5 mmol) in concentrated HCl (75 mL) was heated up to 70 °C, to which SnCl_2_·2H_2_O (9 g, 40 mmol) was added. The mixture was stirred at 70 °C for 30 min and then cooled to 0 °C. Aqueous NH_3_ was added slowly to neutralize HCl, the precipitate was collected by filtration and dissolved in THF. Then the solution was collected by filtration and recrystallized with n-hexane, to give 5,10,15,20-tetrakis(4′-aminophenyl)porphyrin as violet crystal (408 mg) in 40% yield. ^1^H NMR (DMSO-d_6_, 300 MHz): δ (ppm)–2.731 (s, 2H, NH), 5.541 (s, 8H, NH_2_), 7.013 (d, 8H, aromatics), 7.858 (d, 8H, aromatics), 8.874 (s, 8H, H-pyrrole).

### 3.5. Synthesis of 3,6-Diformyl-9-Ethylcarbazole

The compound was prepared according to the literature. [44] A 250-mL three-neck round bottom flask was contained dimethylformamide (DMF, 11 g, 0.15 mol) and cooled to 0 °C. Phosphorus oxychloride (23 g, 0.15 mol) was added dropwise. The solution was allowed to warm up to room temperature and stirred for 1 h, and added a solution of N-ethyl carbazole (2.93 g, 0.015 mol) in 40 mL 1,2-dichloroethane. Then the solution was heated to 90 °C for 48 h. After the reaction completed, it was slowly poured into ice water, and extracted with chloroform for three times. The solution was evaporated under vacuum and the crude product was purified by column chromatography (silica gel, 99:1 *v*/*v* dichloromethane/methanol) provided a white solid (1.8g) in 61% yield. ^1^H NMR (CDCl_3_, 300 MHz): δ(ppm) 1.522 (t, 3H, CH_3_), 4.485 (q, 2H, CH_2_), 7.758 (d, 2H, carbazole), 8.115 (d, 2H, carbazole), 8.683 (s, 2H, carbazole), 10.145 (s, 1H, CH=O).

### 3.6. Synthesis of COF-K

A Pyrex tube (o.d. × i.d. = 10 × 8 mm^2^ and length 200 mm) was charged with 5,10,15,20-tetrakis(4′-aminophenyl) porphyrin (67.5 mg, 0.1 mmol), 3,6-diformyl-9-ethylcarbazole (50 mg, 0.2 mmol), 1 mL of o-dichlorobenzene, 1 mL of n-butanol, and 0.2 mL of 6 M aqueous acetic acid. This mixture was sonicated for 10 min in order to get a homogenous dispersion. The tube was flash frozen at 77 K (liquid N_2_ bath) and degassed by three freeze-pump-thaw cycles and then sealed off. The tube was heated to 120 °C for 3 days. After that, a dark violet precipitate was formed and collected by centrifugation and washed with acetone (10 mL × 3). The powder was purified by Soxhlet extraction with tetrahydrofuran for 3 days and then dried at 120 °C under vacuum for 12 h to give a dark violet powder in 69% (81 mg). Elemental analysis (%) calculated for C 82.34, H 4.85, N 12.81; found: C 78.22, H 5.06, N 12.05.

## 4. Conclusions

In summary, it is the first time that we successfully synthesized a novel one-dimensional COF-K from porphyrin and nonlinear building units. The COF-K is an ordered microporous material with a Type-I N_2_ adsorption isotherm and a narrow pore size distribution for 1.25 nm, but with a relatively weak PXRD pattern owing to lack of π–π interaction from the two-dimensional layers. Different from the previously two-dimensional COFs is that each porphyrin connects four linear building blocks to form two-dimensional networks; each porphyrin links with four nonlinear building blocks to form one-dimensional line in the COF-K. This strategy gives a new direction to build one-dimensional COFs and expands greatly the scope of the emerging materials. The new topology structure may show potential applications in woven materials, flexible conductive materials and super light aerogel.

## Supplementary Materials

The following are available online at https://www.mdpi.com/1420-3049/24/18/3361/s1, Figure S1 SEM image of COF-K. Figure S2 TEM image of COF-K; Figure S3 TGA profile of COF-K; Figure S4 XRD pattern of COF-K and building units. Figure S5 (a) Top view of the simulated two-dimensional AA-eclipsed model structure; (b) Top view of the simulated two-dimensional AB-staggered model structure of COF-K; (c) Side view of the simulated two-dimensional AA-eclipsed model structure of COF-K; (d) Side view of the simulated two-dimensional AB-staggered model structure of COF-K. Figure S6 Experimental PXRD pattern (black curve) and the simulated patterns according to two-dimensional AA-eclipsed (red curve) and AB-staggered (blue curve) stacking models. Figure S7 CO_2_ adsorption-desorption isotherm of COF-K at 273K. Figure S8 ^1^H NMR spectrum of 5,10,15,20-tetrakis(4′-aminophenyl)porphyrin. Figure S9 ^1^H NMR spectrum of 3,6-diformyl-9-ethylcarbazole. Table S1 Unit cell parameters.
