# Supplementary material for: A Novel One-Dimensional Porphyrin-Based Covalent Organic Framework
Source: Molecules. 2019 Sep 16;24(18):3361. doi: 10.3390/molecules24183361 (PMC6767199; doi:10.3390/molecules24183361)
Supplement: Supplementary file 1 [file molecules-24-03361-s001.zip › molecules-585218-SI.pdf]

Supporting Information

A Novel One-dimensional Porphyrin-based Covalent Organic Framework

Miao Zhang [1,2], Ruijin Zheng,[1,2] Ying Ma,[3] Ruiping Chen,[4] Xun Sun,[1,2]* Xuan Sun[3]*

[1]State Key Laboratory of Crystal Materials, Shandong University, Jinan 250100, P. R. China. E-mail: sunxun@sdu.edu.cn
[2]Key Laboratory of Functional Crystal Materials and Device (Shandong University), Ministry of Education, Jinan 250100, China.
[3]Key Laboratory of Colloid and Interface Chemistry, Shandong University, Ministry of Education, Jinan 250100, P. R. China. E-mail: sunxuan@sdu.edu.cn
[4]State Key Laboratory of Structural Chemistry Fujian Institute of Research on the Structure of Matter, Chinese Academy of Sciences Fuzhou, 350002, P. R. China

Contents

Section S1 FE-SEM analysis 3
Section S2 TEM analysis 3
Section S3 Thermogravimetric analysis 4
Section S4 PXRD Pattern of COF-K and building units 4
Section S5 Simulation of two-dimensional structures 5
Section S6 CO_2 adsorption measurement 6
Section S7 Unit cell parameters 7
Section S8 NMR spectra 10

Section S1 FE-SEM analysis

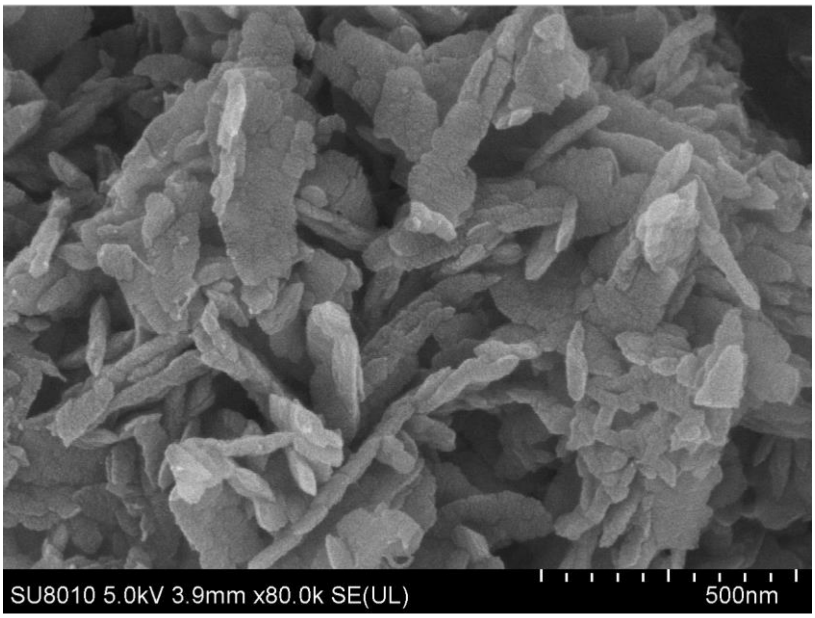

Figure S1. SEM image of COF-K

Section S2 TEM analysis

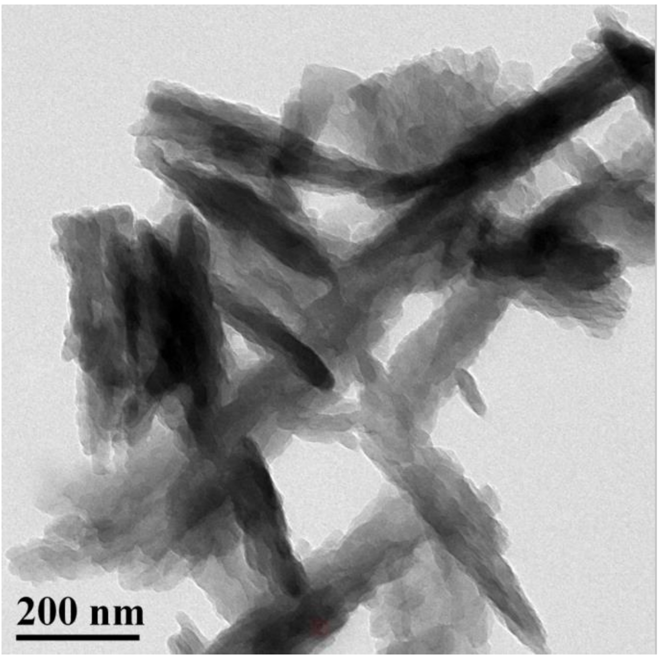

Figure S2. TEM image of COF-K

Section S3 Thermogravimetric analysis

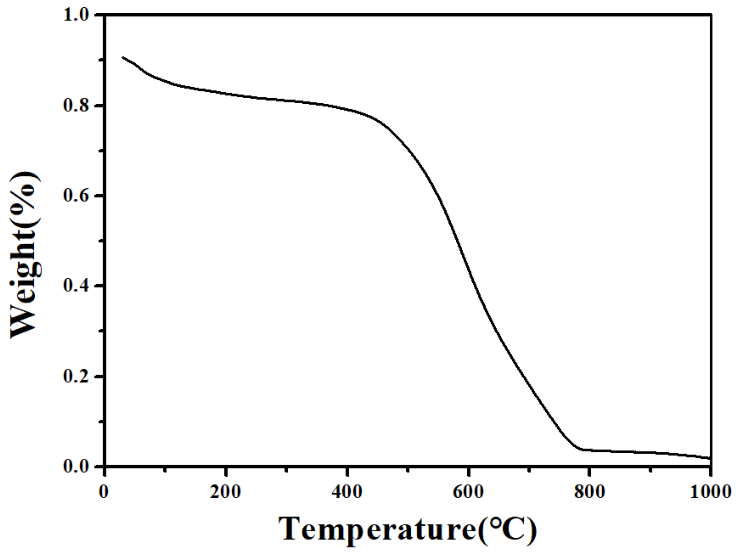

Figure S3. TGA profile of COF-K

Section S4 PXRD Pattern of COF-K and building units

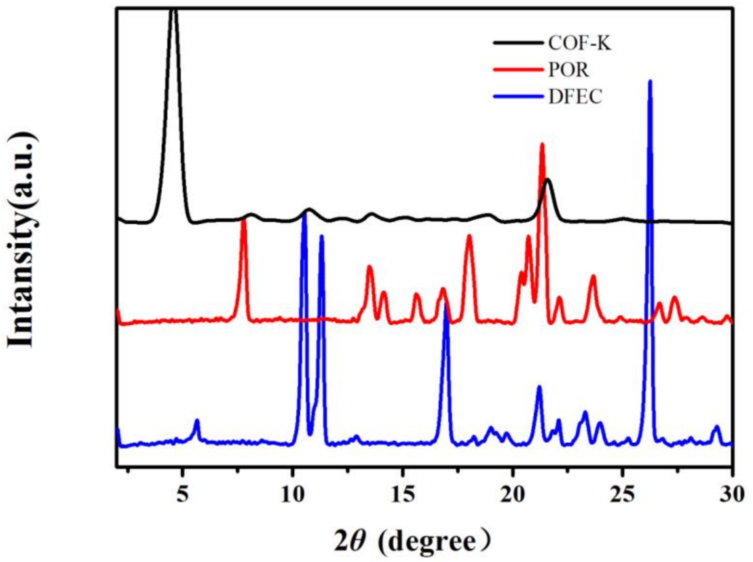

Figure S4. XRD pattern of COF-K and building units.

Section S5 Simulation of two-dimensional structures

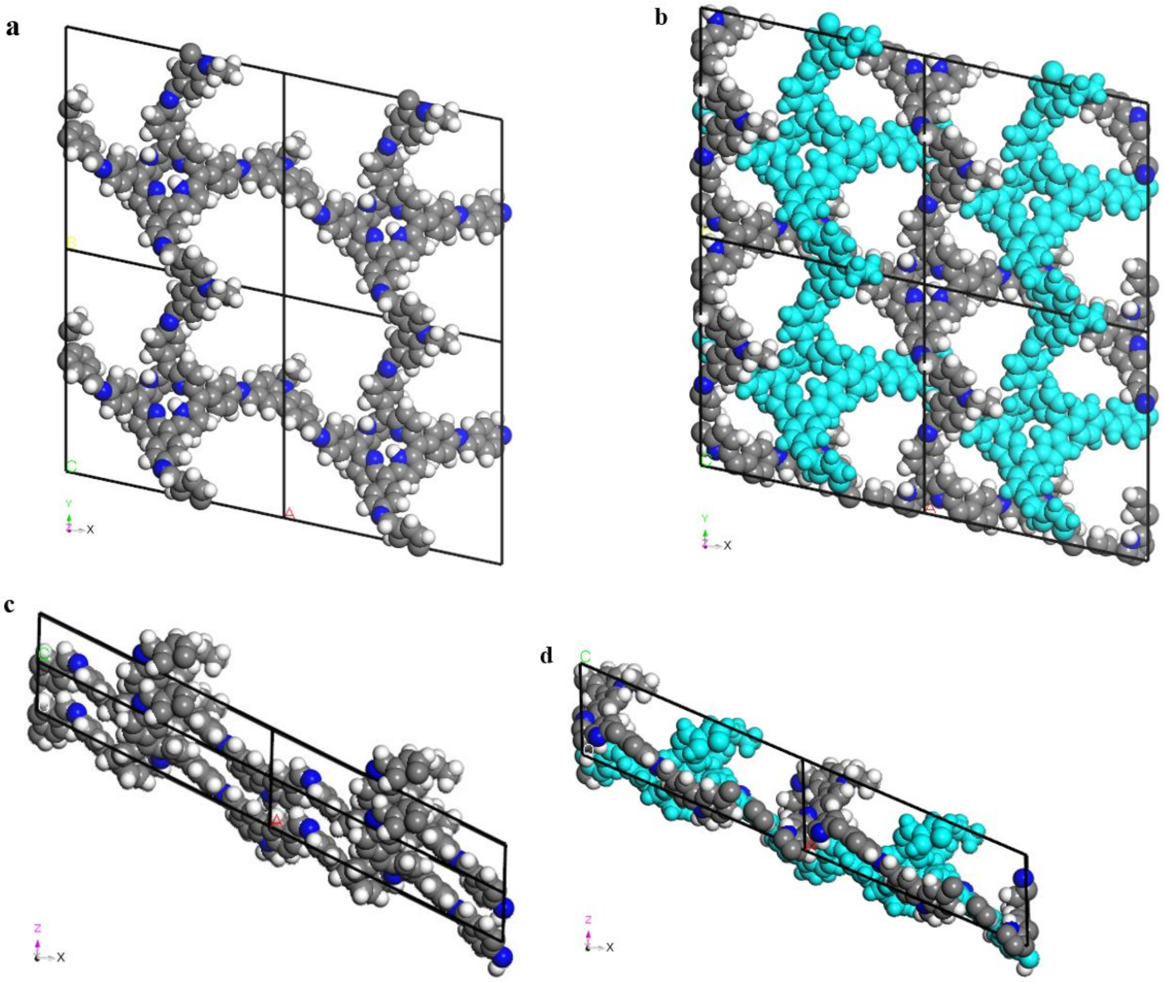

Figure S5. (**a**) Top view of the simulated two-dimensional AA-eclipsed model structure (**b**) Top view of the simulated two-dimensional AB-staggered model structure of COF-K (**c**) Side view of the simulated two-dimensional AA-eclipsed model structure of COF-K (**d**) Side view of the simulated two-dimensional AB-staggered model structure of COF-K.

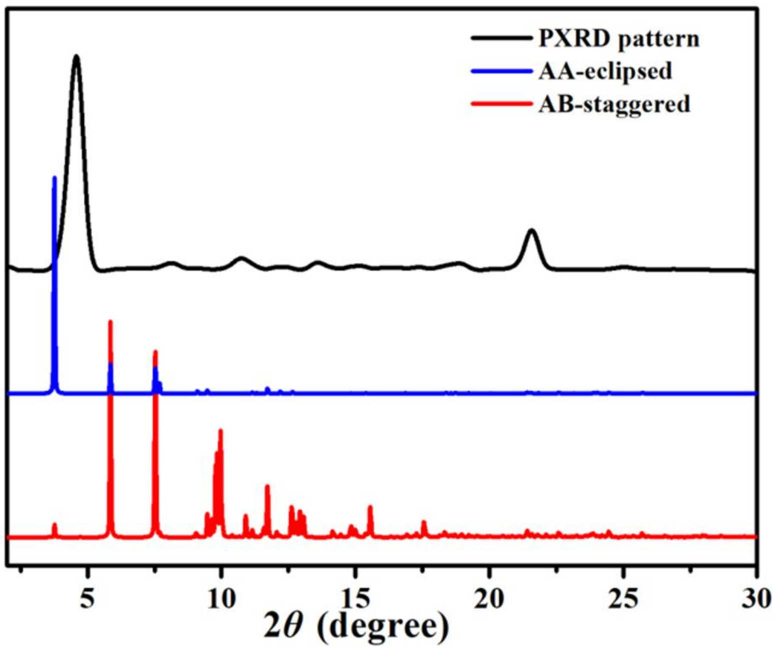

Figure S6. Experimental PXRD pattern (black curve) and the simulated patterns according to two-dimensional AA-eclipsed (red curve) and AB-staggered (blue curve) stacking models.

Section S6 CO_2 adsorption measurement

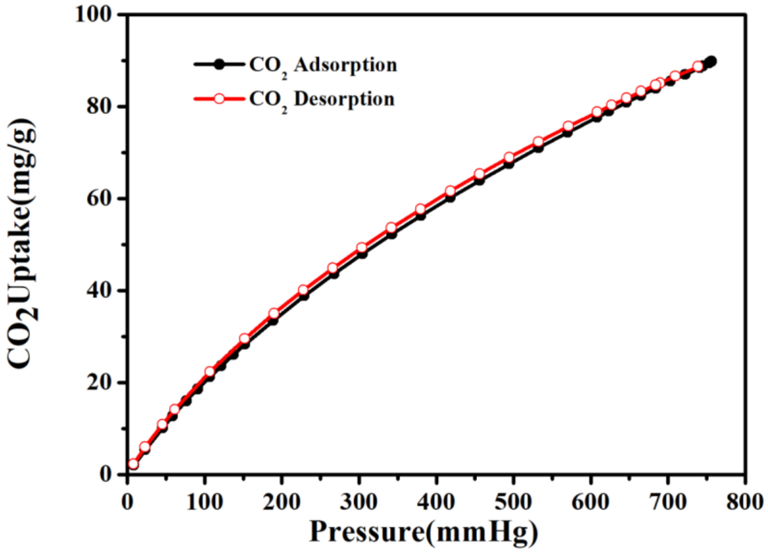

Figure S7. CO_2 adsorption-desorption isotherm of COF-K at 273K.

Section S7 Unit cell parameters

COF-K			
Triclinic P1<br>a=21.6185 Å, b=22.7637 Å, c=4.1495 Å<br>α = 86.0359°, β = 88.8996°,γ =115.2113°			
C1	2.27928	0.52271	-0.21954
C2	2.32762	0.49819	-0.12254
N3	2.38869	0.55093	-0.10051
C4	2.38112	0.60725	-0.15942
C5	2.31178	0.58914	-0.23938
C6	2.31495	0.43285	-0.04307
C7	2.35147	0.34626	0.1632
C8	2.41193	0.34227	0.17837
C9	2.46339	0.40492	0.03422
N10	2.43497	0.4469	-0.04752
C11	2.36651	0.41151	0.01754
C12	2.53278	0.41813	-0.02376
C13	2.58412	0.48088	-0.12084
C14	2.65319	0.4986	-0.23509
C15	2.68557	0.56495	-0.29066
C16	2.63756	0.58989	-0.20594
N17	2.57667	0.53737	-0.11612
C18	2.65036	0.65542	-0.19285
C19	2.50197	0.68337	-0.10451
N20	2.53022	0.64127	-0.15638
C21	2.59872	0.67665	-0.13865
C22	2.61393	0.74191	-0.05358
C23	2.5536	0.74608	-0.02758
C24	2.43256	0.67017	-0.13387
C25	2.24343	0.38439	-0.01425
C26	2.41249	0.72398	-0.13668
C27	2.55275	0.36423	0.01728
C28	2.72183	0.70412	-0.22648
C29	2.35317	0.71756	0.05529
C30	2.3343	0.76824	0.05977
C31	2.37471	0.82867	-0.12811
C32	2.43447	0.83589	-0.31673
C33	2.45225	0.78461	-0.32526

C34	2.7408	0.76392	-0.43188
C35	2.80751	0.8107	-0.46686
C36	2.85955	0.79997	-0.29629
C37	2.84136	0.74027	-0.09395
C38	2.77429	0.69384	-0.05791
C39	2.61224	0.37072	0.19048
C40	2.63095	0.31997	0.23709
C41	2.59048	0.25942	0.11143
C42	2.53072	0.25226	-0.05864
C43	2.51282	0.30349	-0.10829
C44	2.22397	0.32447	-0.16242
C45	2.15723	0.27785	-0.13917
C46	2.10567	0.28899	0.03515
C47	2.12431	0.34894	0.17948
C48	2.19148	0.39519	0.15792
N49	2.92797	0.8433	-0.31922
C50	2.94871	0.90249	-0.4524
C51	3.02023	0.94718	-0.48716
C52	3.03978	1.01093	-0.64405
C53	3.10749	1.05615	-0.67677
C54	3.15627	1.0362	-0.54922
C55	3.13786	0.9713	-0.40338
C56	3.06982	0.92739	-0.36857
C57	3.20066	0.96651	-0.3314
C58	3.25402	1.02893	-0.42554
N59	3.22617	1.07051	-0.54646
C60	3.32259	1.04104	-0.40733
C61	3.33729	0.98917	-0.30865
C62	3.28517	0.92595	-0.2286
C63	3.21674	0.91514	-0.23109
C64	3.26511	1.13731	-0.68486
C65	3.23835	1.18342	-0.53883
C66	3.30245	0.87136	-0.18884
N67	3.04145	0.24187	-0.01844
C68	3.02089	0.18312	-0.10319
C69	2.94957	0.13826	-0.09226
C70	2.9312	0.07629	-0.21439
C71	2.86368	0.03136	-0.21584
C72	2.81415	0.05005	-0.09247
C73	2.83131	0.11282	0.02461
C74	2.89924	0.15653	0.02743
C75	2.76763	0.11661	0.09701

C76	2.71508	0.05594	0.02527
N77	2.74416	0.01595	-0.07642
C78	2.64639	0.0441	0.04365
C79	2.63099	0.09487	0.11789
C80	2.68239	0.15644	0.17869
C81	2.75065	0.16652	0.17934
C82	2.66543	0.21105	0.19206
N83	2.60501	0.20583	0.13622
C84	2.70878	-0.0475	-0.2052
C85	2.7264	-0.09909	-0.01162
N86	1.3557	0.88593	-0.05653
H87	2.22578	0.49272	-0.26539
H88	2.43517	0.54933	-0.06381
H89	2.28953	0.62321	-0.30154
H90	2.30092	0.30832	0.24441
H91	2.42094	0.30068	0.28018
H92	2.67536	0.46435	-0.26764
H93	2.73877	0.5946	-0.38101
H94	2.5303	0.53912	-0.06994
H95	2.66449	0.77959	-0.01512
H96	2.54476	0.78767	0.04195
H97	2.32313	0.67168	0.21833
H98	2.28976	0.76246	0.23025
H99	2.46479	0.88285	-0.47022
H100	2.49709	0.78996	-0.49061
H101	2.701	0.77163	-0.57803
H102	2.82006	0.85485	-0.64863
H103	2.88203	0.73273	0.04485
H104	2.76093	0.64884	0.11659
H105	2.64261	0.4169	0.3038
H106	2.67592	0.32638	0.39103
H107	2.50051	0.20522	-0.1646
H108	2.46776	0.29791	-0.2585
H109	2.26337	0.31661	-0.31292
H110	2.14426	0.23347	-0.27825
H111	2.08399	0.35692	0.32056
H112	2.20536	0.44057	0.28725
H113	2.91299	0.92333	-0.54682
H114	2.99991	1.0246	-0.74627
H115	3.12168	1.10551	-0.80226
H116	3.05253	0.87707	-0.25115
H117	3.36396	1.08926	-0.48556

H118	3.39007	0.99463	-0.30599
H119	3.17623	0.86567	-0.17181
H120	3.26616	1.14017	-0.9845
H121	3.31831	1.15094	-0.61099
H122	3.19454	1.15675	-0.34268
H123	3.22055	1.20764	-0.75517
H124	3.2779	1.22186	-0.39691
H125	3.25841	0.82241	-0.15013
H126	3.05678	0.1629	-0.19295
H127	2.97181	0.06417	-0.3192
H128	2.85012	-0.01615	-0.32101
H129	2.91585	0.20562	0.11628
H130	2.60588	-0.00276	-0.01632
H131	2.57845	0.09009	0.11641
H132	2.79061	0.21466	0.2257
H133	2.70919	0.25876	0.22495
H134	2.72152	-0.04266	-0.49913
H135	2.65352	-0.06094	-0.17637
H136	2.73532	-0.12964	-0.20621
H137	2.68584	-0.13158	0.1895
H138	2.77401	-0.07638	0.13262

Section S8 NMR spectra

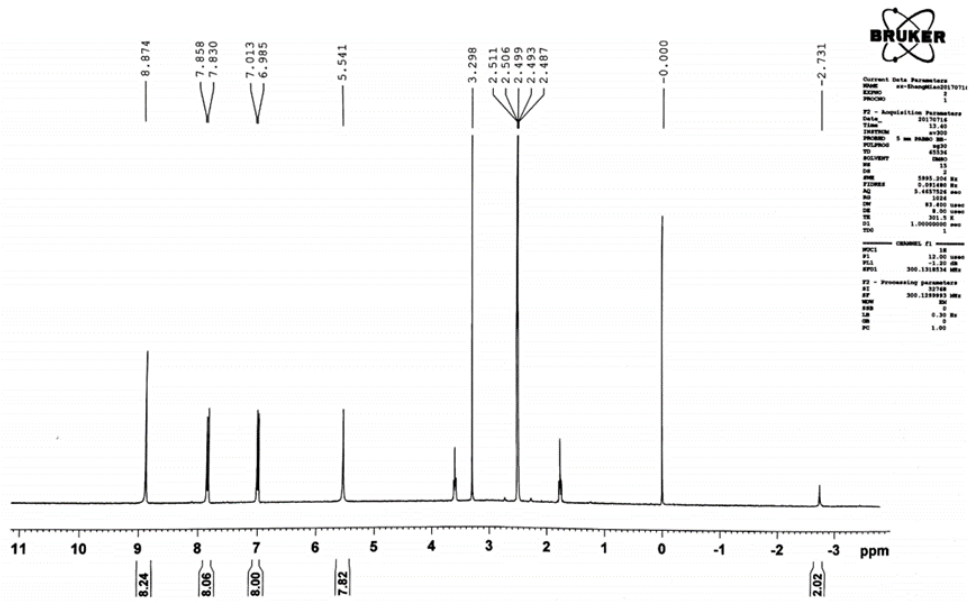

Figure S8. ^{1}H NMR spectrum of 5,10,15,20-tetrakis(4′-aminophenyl)porphyrin

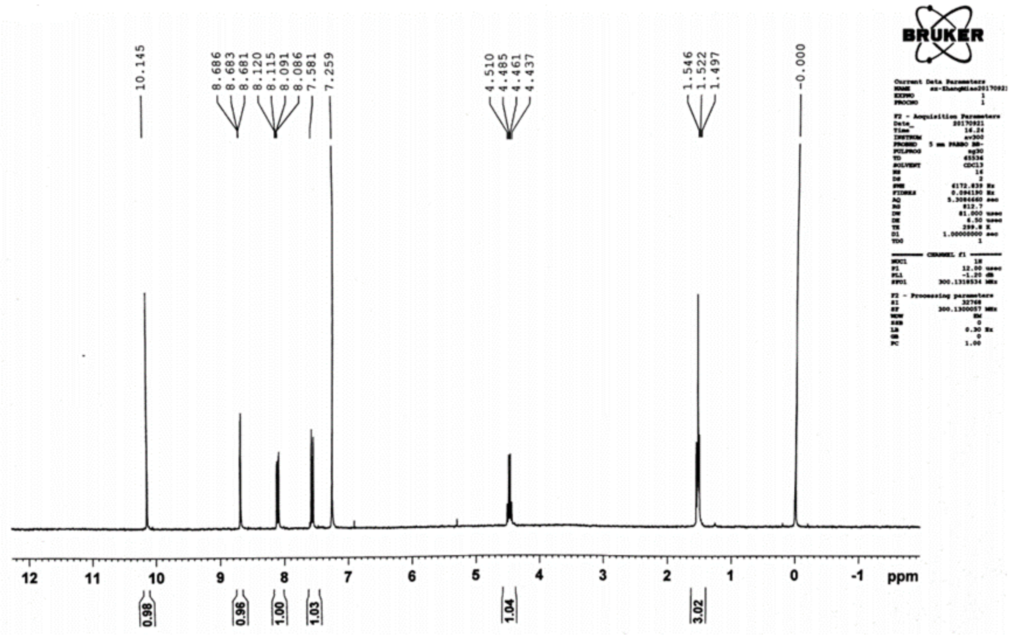

Figure S9. ^{1}H NMR spectrum of 3,6-diformyl-9-ethylcarbazole
